# Invasion of *Spartina alterniflora* on *Zostera japonica* enhances the abundances of bacteria by absolute quantification sequencing analysis

**DOI:** 10.1002/ece3.8939

**Published:** 2022-05-19

**Authors:** Zenglei Song, Yanyu Sun, Pengyuan Liu, Yibo Wang, Yanyan Huang, Yan Gao, Xiaoke Hu

**Affiliations:** ^1^ Key laboratory of Coastal Biology and Bioresource Utilization Yantai Institute of Costal Zone Research Chinese Academy of Sciences Yantai China; ^2^ Laboratory for Marine Biology and Biotechnology Qingdao National Laboratory for Marine Science and Technology Qingdao China; ^3^ University of Chinese Academy of Sciences Beijing China; ^4^ Marine Science Research Institute of Shandong Province National Oceanographic Center of Qingdao Qingdao China

**Keywords:** absolute quantification 16S rRNA sequencing, plant invasion, sediment bacterial community, the Yellow River Estuary

## Abstract

Plant invasion can alter soil organic matter composition and indirectly impact estuary ecology; therefore, it is paramount to understand how plant invasion influences the bacterial community. Here, we present an absolute quantification 16S rRNA gene sequencing to investigate the bacterial communities that were collected from *Zostera japonica* and *Spartina alterniflora* covered areas and *Z*. *japonica* degradation areas in the Yellow River Estuary. Our data revealed that the absolute quantity of bacteria in the surface layer was significantly (*p *< .05) higher than that in the bottom and degradation areas. Following the invasion of *S*. *alterniflora*, the abundances of Bacteroidia, Acidimicrobiaceae, and Dehalococcoidaceaewere enriched in the *S*. *alterniflora* sediment. In addition, variations in the composition of sediment bacterial communities at the phylum level were the most intimately related to total organic carbon (TOC), and the content of heavy metals could reduce the abundance of bacteria. This study provided some information to understand the effects of *S*. *alterniflora* invasion on *Z*. *japonica* from the perspective of microbiome level.

## Graphical abstract

The absolute quantity of surface layer bacteria was higher than the other area. *Spartina alterniflora* invasion increased the bacterial community abundance. The Sulfate‐Reducing Bacteria and Flavobacteriaceae are dominant bacteria in seagrass beds.

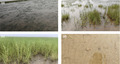

## 1 INTRODUCTION

*Zostera japonica* is one of the most extensively distributed seagrass species on earth (Shafer et al., 2014). Seagrasses filter nutrients and pollutants from estuaries and coastal water, purify water qualities, stabilize sediment, protect biodiversity, and participate in the recycling of materials and carbon fixation. In addition, it is also the habitat, nursery, and food sources of many marine animals (Lamb et al., 2017). Although it is not taller than *Zostera marina*, the shoot density of *Z*. *japonica* is much higher than that of *Z*. *marina* (Sugimoto et al., 2017). However, seagrass meadows are fragile and vulnerable to threat around the world, and their degradation is accelerating (Short et al., 2011; Waycott et al., 2009). Up to 29% of the world's seagrass has wholly disappeared, and the rapid decline continues unabated, with approximately 7% disappearing from the planet every year (Waycott et al., 2009). The distribution of *Z*. *japonica* in China has been recently reported based on large‐scale surveys (Zhang et al., 2015; Zheng et al., 2013). Due to swift declines resulting from increasingly harsh artificial and habitat destruction, larger areas of *Z*. *japonica* meadows are very rare now (Abe et al., 2003, 2009; Lee et al., 2005; Mach et al., 2014; Zhang et al., 2015). In 2015, a vast *Z*. *japonica* bed with an area ca. 1000 ha was found in the Yellow River Estuary (Shandong, China) (Zhou et al., 2016).

To protect the seacoast by reducing coastal tidal erosion, *S*. *alterniflora*, which originates from the Atlantic and Gulf Coasts of North America, was introduced in China in 1979 (Cui et al., 2017). Since then, *S*. *alterniflora* has spread and invaded in native environments aggressively, occupying the habitats by ejecting native plant species (Nie et al., 2009). Fields observations showed that *Z*. *japonica* was continuously distributed on the seaward side of the habitat and mixed with *Spartina alterniflora* in the Yellow River Estuary (Shandong, China) (Zhou et al., 2016). The invasion of *S*. *alterniflora* not only threatens the biodiversity of native ecosystems but also shifts ecosystem processes, roles, and services, causing variation in the carbon, nitrogen, phosphorus, and sulfur cycles in the invaded ecosystem (Ehrenfeld, 2003; Liao et al., 2008; Zhou et al., 2007). Field‐controlled experiments showed that the density effects of *S*. *alterniflora* significantly inhibited the *Z*. *japonica* (Ma et al., 2020). Nonnative plant species invasion has a significant influence on coastal ecosystems and modifies ecosystem functions through a huge variety of mechanisms.*S*. *alterniflora* invasion could alter the composition of the soil microbial community in coastal salt marshes within a short‐term invasion history (Zhang, Bai, et al., 2019). Mangrove wetlands in China have severely suffered from invasions of *S*. *alterniflora* resulting in habitat modification and loss. Previous studies have indicated the impact of *S*. *alterniflora* invasion on mangrove ecosystems, including changes in the biomass of mangrove forests (Wang et al., 2014; Zhang et al., 2012), microeukaryotic diversity (Yu et al., 2014), the composition of ammonia oxidizers (Hawkes et al., 2005; Zhang et al., 2011), and the response of the structure of the microbial community of mangrove soil to *S*. *alterniflora* invasion (Zheng et al., 2019).

Previous studies have shown that plant species are key drivers of rhizosphere microbiome composition and functioning (Marschner et al., 2001; Mendes et al., 2018). For example, the *Alnus trabeculosa* increased the soil bacterial diversity in the invaded regions (Chen et al., 2016). Another invasive plant, *Mikania micrantha*, has a distinct bacterial community structure that is clearly separated from the native plants and the bulk soil (Yin et al., 2020). However, the impacts of *S*. *alterniflora* invasion on sediment bacterial abundance, diversity, and community composition in contrast to *Z*. *japonica* remain uncertain. We hypothesized that on the one hand, *S*. *alterniflora* invasion may change the environmental bacterial communities in sediment; on the other hand, there are some dominant groups that are better adapted to the specific environment, to realize the invasion on local plants. To test these hypotheses, the absolute quantification sequencing of the bacterial 16S rRNA gene was undertaken, to analyze variations in sediment bacterial abundance, diversity, and community composition. Total carbon (TC), total nitrogen (TN), total organic carbon (TOC), total organic nitrogen (TON), and some heavy metals in sediment (including Pb, Cr, Co, Ni, Cu, Zn, As, Cd, Al, Ti, V, Mn, and Fe) were also examined in this study.

## 2 MATERIALS AND METHODS

### 2.1 Sampling site description

**FIGURE 1:**
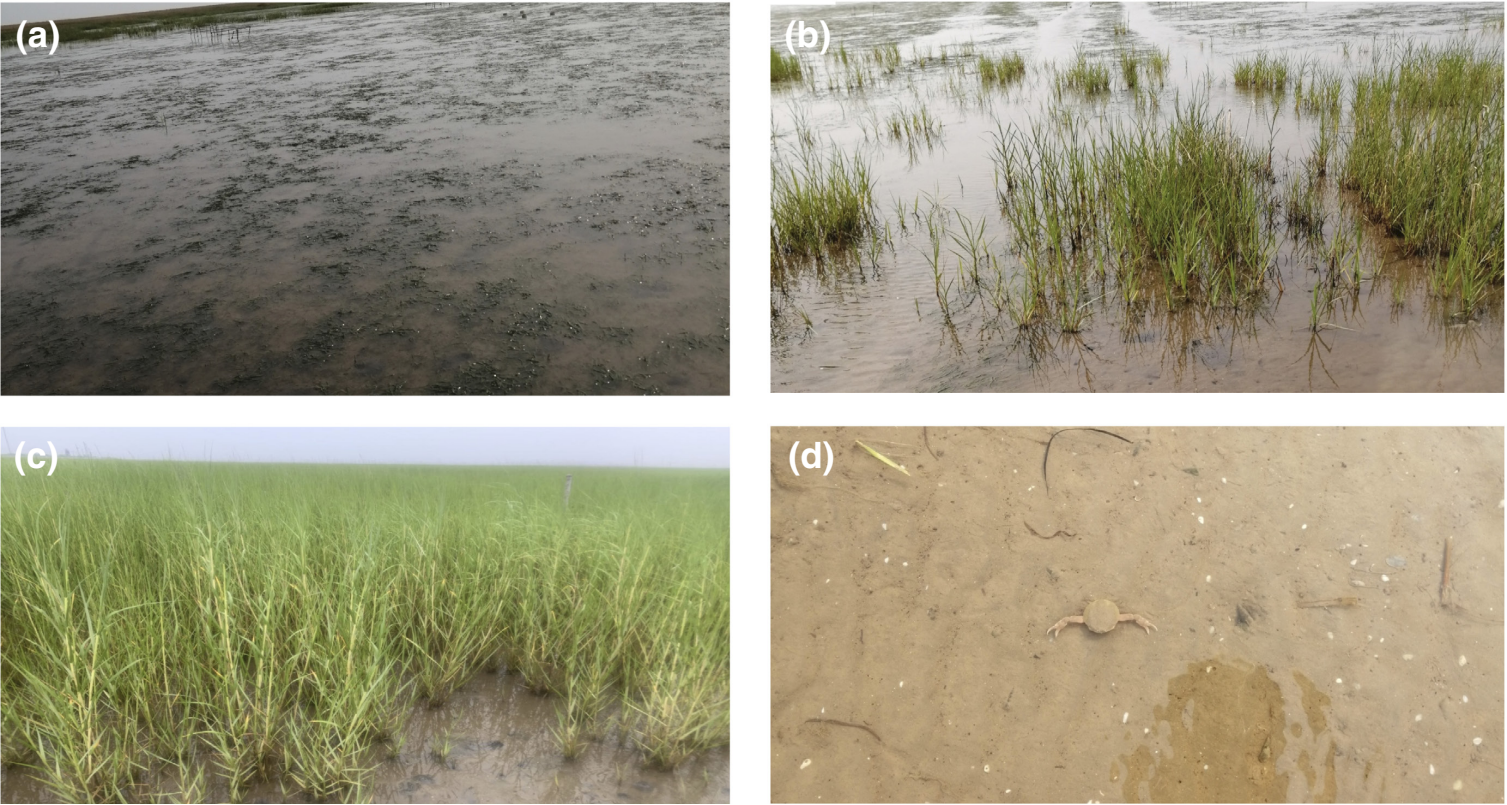
The field control experiments photos. (Note a: *Z*. *japonica* areas; b: mixed areas of *Z*. *japonica* and *S*. *alterniflora*; c: *S*. *alterniflora* areas; d: Degradation areas)

This study was carried out in the Yellow River Estuary of Shandong Province (37°51′N, 119°6′E). This area is characterized by a temperate continental monsoon climate with four distinct seasons (Jiang et al., 2013). The annual mean temperature ranges from 11.5 to 12.4°C, the annual rainfall is approximately 600 mm, and pan evaporation exceeds 1500 mm (Kong et al., 2015). Approximately 70%–80% of the total annual precipitation takes place in summer. The *Z*. *japonica* bed in this study area is the largest *Z*. *japonica* bed found in China, and it is also the largest single species of seagrass bed (Zhou et al., 2016). In addition, *Z*. *japonica* was continuously distributed on the seaward side of the habitat and mixed with *Spartina alterniflora* (Figure 1 (this figure could be considered for the publication cover)).

### 2.2 Sampling design and procedures

Field sample collection was carried out in June 2019. Sediment was collected at low tide. To see the difference between the bacterial communities insediments of *Z*. *japonica* and *S*. *alterniflora*, we selected mixed areas of *Z*. *japonica* and *S*. *alterniflora* with a 50m interval. Sediment samples were randomly collected from 0 to 20 cm depths in each area using a sediment core sampler (diameter of 2.0 cm), of which 0–5 cm was used as the surface sample of the sediment, and of which 15–20 cm was used as the bottom sample of the sediment (Souri et al., 2020). In addition, the sample sediment of *Z*. *japonica* was degraded as the control (Figure 1). Thus, 15 sediment samples containing 3 samples of the *Z*. *japonica* surface, 3 samples of the *Z*. *japonica* bottom, 4 samples of the *S*. *alterniflora* surface, 4 samples of the *S*. *alterniflora* bottom, and one sample of degradation (unvegetated area) were obtained. All the samples were immediately placed on ice and transported to the laboratory, where they were stored at −80°C until physicochemical properties measurement and DNA extraction.

### 2.3 Physicochemical properties measurements

In the laboratory, each sample was homogenized entirely after removing the plant roots and other debris. Total carbon (TC), total nitrogen (TN), total organic carbon (TOC), and total organic nitrogen (TON) were measured using a small injection elemental analyzer (Vario Micro cube). Before TOC/TON analysis, samples were acidified with dilute HCl (1 mol/L) to remove carbonates and then subsequently washed with deionized water three times before drying overnight at 60°C. The trace element concentrations were detected by an inductively coupled plasma mass spectrometer (ELAN DRC II, PerkinElmer Ltd.). Additionally, a Malvern Mastersizer 2000 laser diffractometer that can analyze particle sizes between 0.02 and 2000 μm to analyze the particle sizes of the sediment samples was used. Meanwhile, we determined the percentages of the following three size groups: <4 μm (clay), 4– 63 μm (silt), and >63 μm (sand) (Jiang et al., 2017).

### 2.4 Absolute quantification 16S‐ rRNA sequencing

A total of 15 sediment samples collected from the five groups (*Z*. *japonica* surface (ZS), *Z*. *japonica* bottom (ZB), *S*. *alterniflora* surface (SS), *S*. *alterniflora* bottom (SB), and degradation (DE)) were sent to Genesky Biotechnologies Inc., Shanghai, 201315 (China) for absolute quantification of 16S rRNA amplicon sequencing by MiSeq. Briefly, total genomic DNA was extracted from 0.5 g (dry weight) of each sample using the Fast DNA^®^ SPIN Kit for Soil (MP Biomedicals) according to the manufacturer's specifications. The integrity of genomic DNA was detected through agarose gel electrophoresis, and the concentration and purity of genomic DNA were detected through the Nanodrop 2000 and Qubit3.0 Spectrophotometer. Multiple spike‐ins with identical conserved regions to natural 16S rRNA genes and variable regions replaced by random sequence with ~40% GC content was artificially synthesized. Then, appropriate proportion of spike‐ins mixture with known gradient copy numbers were added to the sample DNA. The V4–V5 hypervariable regions of the 16S rRNA gene and spike‐ins were amplified with the primers 515F (5′‐GTGCCAGCMGCCGCGG‐3′) and 907R (5′‐CCGTCAATTCMTTTRAGTTT‐3′) (Biddle et al., 2008) and the PCR conditions were used: an initial denaturation at 94°C for 2 min; 25 cycles at 94°C for 30 s, 55°C for 30 s and 72°C for 60 s; and a final extension at 72°C for 10 min. The PCR products were analyzed using 1.2% agarose gel electrophoresis with equimolar concentrations and were purified with QIAquick Gel Extraction Kit (QIAGEN). Then, the library quality was assessed on Qubit@ 2.0 Fluorometer (Thermo Scientific) and AgilentBioanalyzer 2100 system. Finally, the library sequenced using Illumina NovaSeq 6000 sequencer.

### 2.5 Illumina reads data processing and analysis

The raw sequencing data were processed in QIIME2 (Bolyen et al., 2019). The adaptor and primer sequences were trimmed using the cutadapt plugin. DADA2 plugin was used for quality control and to identify amplicon sequence variants (ASVs)(Callahan et al., 2016). Taxonomic assignments of ASVs representative sequences were performed with confidence threshold 0.8 by a pre‐trained Naïve Bayes classifier which was trained on the RDP (version 11.5). Then, the spike‐in sequences were filtered out, and the reads were counted. Standard curve for each sample was generated based the read‐counts versus spike‐in copy number, and the absolute copy number of each ASV in each sample was calculated by using the read‐counts of the corresponding ASV. As the spike‐in sequence is not a component of the sample flora, the spike‐in sequence needs to be removed in the subsequent analysis (Jiang et al., 2019).

### 2.6 Statistical analyses

The data were analyzed using IBM SPSS 28.0 (IBM Corporation). The diversity indices are related to the sequencing depth, so the ASV taxonomy table with spike‐in sequence deleted and copy number not calculated is selected to calculate the diversity indices. They were calculated and statistically examined by non‐parametric statistics analysis (Kruskal‐Wallis tests). The sediment physicochemical properties of different groups (the degradation group was not included) meet the assumptions of ANOVA. They were calculated and statistically examined by one‐way analysis of variance (ANOVA). The significance of differences between group means (the degradation group was not included) was evaluated with Tukey's honest significant difference test at *p* < .05. Spearmancorrelation analysis was used to evaluate the absolute abundances of bacterial phyla with physicochemical properties.PICRUSt2 (version 2.3.0) analysis tools (Douglas et al., 2020) were used to predict and analyze their species function. Non‐metric multidimensional scaling (NMDS), Spearman correlation, and redundancy analysis (RDA) (Fathollahi‐Fard et al., 2020) were implemented by R using the vegan packages (version 2.4.5). To identify potentially discriminating taxa among the four groups (excluding the degradation group), LEfSe was applied (Zhang et al., 2013). First, the nonparametric Kruskal‐Wallis sum rank test was used to detect the differential abundant features (genera, families, classes, phyla) among four groups. Then, based on the significantly different species obtained, a paired Wilcoxon rank sum test was used to analyze the difference between subgroups. Finally, the effective size of each differential abundant feature was estimated using linear discriminant analysis. All‐against‐all classes were compared (most stringent) and a value of 2.0 of the logarithmic linear discriminant analysis score was chosen as the threshold for discriminative features.

## 3 RESULTS

### 3.1 Physicochemical properties of sediment samples

Sediments in all samples were pale yellow in color, with a few macrozoobenthic organisms visible. The substrate of the Yellow River Estuary is mainly slit (48.7%–60.2%) and sand (35.2%–47.5%). The majority of trace element concentrations did not exhibit significant (*p *< .05) spatial heterogeneity (Table 1). Only the contents of Pb and V in the ZS sample were significantly (*p *< .05) lower than those of the other groups (the DE group was not included). The contents of Cr, Co, Ni, Cu, Zn, Al, and Fe in the DE sample were higher than those of the other groups. In addition, the concentrations of Cr, Zn, Al, and Fe showed a similar trend of DE > SB > ZB > SS > ZS among sites (Table 1).

**TABLE 1:** Spatial difference in sediment physicochemical properties

Physicochemical properties	Groups				Source of variation groups	Groups
	ZS (*n *= 3)	ZB (*n *= 3)	SS (*n *= 4)	SB (*n *= 4)		DE (*n *= 1)
TON%	0.02 ± 0.00^a^	0.02 ± 0.00^a^	0.03 ± 0.01^a^	0.02 ± 0.00^a^	n.s.	0.02
TOC%	0.16 ± 0.03^a^	0.15 ± 0.01^a^	0.29 ± 0.07^a^	0.17 ± 0.03^a^	n.s.	0.16
TN%	0.02 ± 0.00^a^	0.02 ± 0.00^a^	0.03 ± 0.01^a^	0.02 ± 0.00^a^	n.s.	0.02
TC%	1.23 ± 0.03^a^	1.21 ± 0.01^a^	1.37 ± 0.09^a^	1.24 ± 0.04^a^	n.s.	1.27
Pb (mg/kg)	11.34 ± 1.45^b^	14.89 ± 0.44^ab^	16.17 ± 0.47^a^	16.41 ± 0.77^a^	**	15.36
Cr (mg/kg)	4.40 ± 0.51^a^	5.25 ± 0.79^a^	4.46 ± 0.28^a^	5.47 ± 0.59^a^	n.s.	9.21
Co (mg/kg)	4.06 ± 0.55^a^	4.93 ± 0.36^a^	4.98 ± 0.18^a^	4.91 ± 0.38^a^	n.s.	6.56
Ni (mg/kg)	12.32 ± 1.57^a^	16.20 ± 0.97^a^	14.98 ± 0.59^a^	16.18 ± 1.11^a^	n.s.	19.94
Cu (mg/kg)	9.51 ± 1.12^a^	11.07 ± 0.77^a^	11.57 ± 0.80^a^	12.05 ± 0.74^a^	n.s.	16.03
Zn (mg/kg)	22.50 ± 3.40^a^	29.33 ± 2.97^a^	26.00 ± 1.46^a^	29.95 ± 2.37^a^	n.s.	41.41
As (mg/kg)	8.75 ± 1.37^a^	11.47 ± 0.09^a^	11.61 ± 0.85^a^	11.19 ± 0.24^a^	n.s.	10.4
Cd (mg/kg)	0.26 ± 0.03^a^	0.31 ± 0.01^a^	0.31 ± 0.02^a^	0.29 ± 0.01^a^	n.s.	0.31
Al (mg/kg)	2824 ± 282^a^	3551 ± 379^a^	3572± 115^a^	3917 ± 576^a^	n.s.	5430.113
Ti (mg/kg)	7153 ± 485^a^	9735 ± 730^a^	8630 ± 347 ^a^	11,710 ± 1963^a^	n.s.	12,376.23
V (mg/kg)	24.46 ± 3.04^b^	32.32 ± 1.13^a^	30.74 ± 1.48^a^	32.17 ± 1.46^a^	*	33.77
Mn (mg/kg)	412.47 ± 57.13^a^	585.04 ± 81.16^a^	559.32 ± 97.48^a^	579.33 ± 87.75^a^	n.s.	489.65
Fe (mg/kg)	4948 ± 609^a^	6810 ± 659^a^	5823 ± 203^a^	6897 ± 701^a^	n.s.	8978.47
D50(um)	50.41 ± 0.83^a^	50.89 ± 0.62^a^	61.12 ± 4.31^a^	53.26 ± 4.55^a^	n.s.	49.29

Note

Data are expressed as mean ± SE (DE group was not included). n.s., not significant; *p < .05; **p < .01(one‐way ANOVA). Different superscript lower case letters indicate statistically significant differences at the α = .05 level among the sediment groups, using Tukey's honestly significant difference test (DE group was not included).

Abbreviations: D50, the median diameter or the medium value of the particle size distribution; DE, degradation;Pb (Cr, Co, Ni, Cu, Zn, As, Cd, Al, Ti, V, Mn, Fe), the heavy mental concentrations in sediment; SB, *S*. *alterniflora* bottom; SS, *S*. *alterniflora* surface; TC, total carbon; TN, total nitrogen; TOC, total organic carbon; TON, total organic nitrogen; ZB, *Z*. *japonica* bottom; ZS, *Z*. *japonica* surface.

### 3.2 Absolute quantification of sediment bacterial community

A total of 3,619,871 raw sequence for 15 samples (5 groups) were obtained. After the data quality filtering, noise reduction, splicing and de chimerism, a total of 2,898,018 sequence were remained for subsequent analysis. The number of generated ASVs in each sample varied from 5314 to 6302 and was further classified by the RDP database. The Venn diagram of the generated ASVs from different groups (Appendix S1: Figure A1) showed that 1594 ASVs were shared among all the groups. Rarefaction curves (Appendix S1: Figure A2) showed that near‐complete of the bacterial diversity had been covered. Analysis of the diversity of a single sample (alpha diversity) can reflect the community richness and diversity. The Sobs, Chao, and ACE indices represent community richness, and the Shannon and Simpson indices represent community diversity. These indices (including coverage index) of the five group sediments are all calculated and shown in Table 2. The results showed that the degradation sediment had the lowest Simpson index scores, while this group also had a higher Shannon index than the other four groups (Table 2). The above results indicated that although there was no significant difference in community richness between groups, the community diversity in the degradation area was higher than that of the other plant covered groups (Table 2).

**TABLE 2:** Bacterial community diversity and richness of samples

Diversity Index	Groups					*p* Value
	ZS (*n* = 3)	ZB (*n* = 3)	SS (*n* = 4)	SB (*n* = 4)	DE (*n* = 1)	
Sobs	4351	4110	4157	4305	3972	.538
Chao1	4406	4140	4213	4368	4032	.639
ACE	4390	4130	4191	4340	3999	.538
Shannon	7.41	7.49	7.41	7.46	7.51	.407
Simpson	0.0016	0.0013	0.0015	0.0015	0.0012	.118
Coverage	0.9989	0.9991	0.9990	0.9990	0.9991	.463

Note

Data are expressed as medians (DE group was not included). *p* value (Kruskal‐Wallis test).

Abbreviations: DE, degradation; SB, *S*. *alterniflora* bottom; SS, *S*. *alterniflora* surface; ZB, *Z*. *japonica* bottom; ZS, *Z*. *japonica* surface.

**FIGURE 2:**
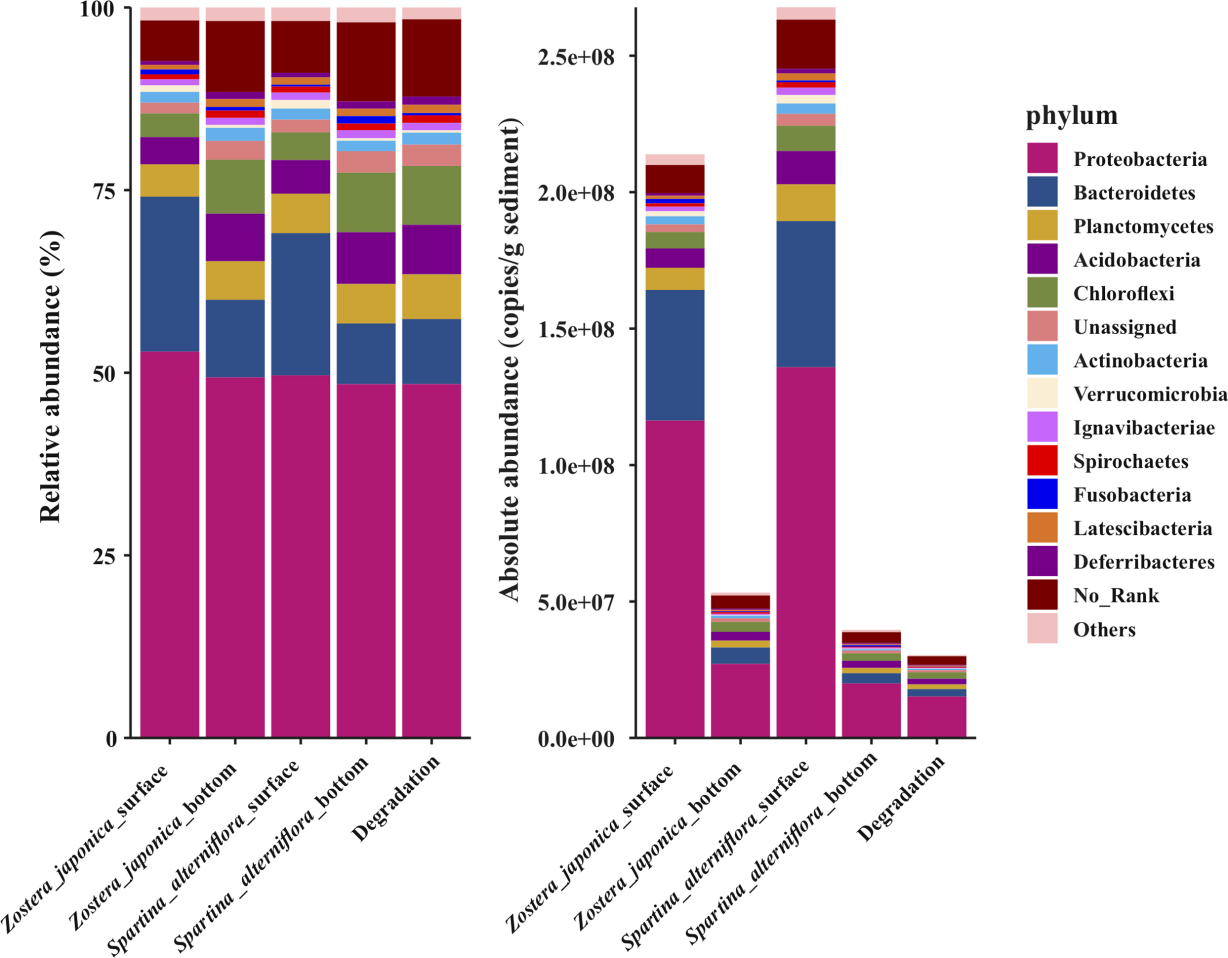
The relative and absolute abundances of the major bacteria at the phylum level. Absolute abundances (16S rRNA gene copies per g of sediment) and relative abundances (%) of the major bacterial phyla present in all the sediment samples

**FIGURE 3:**
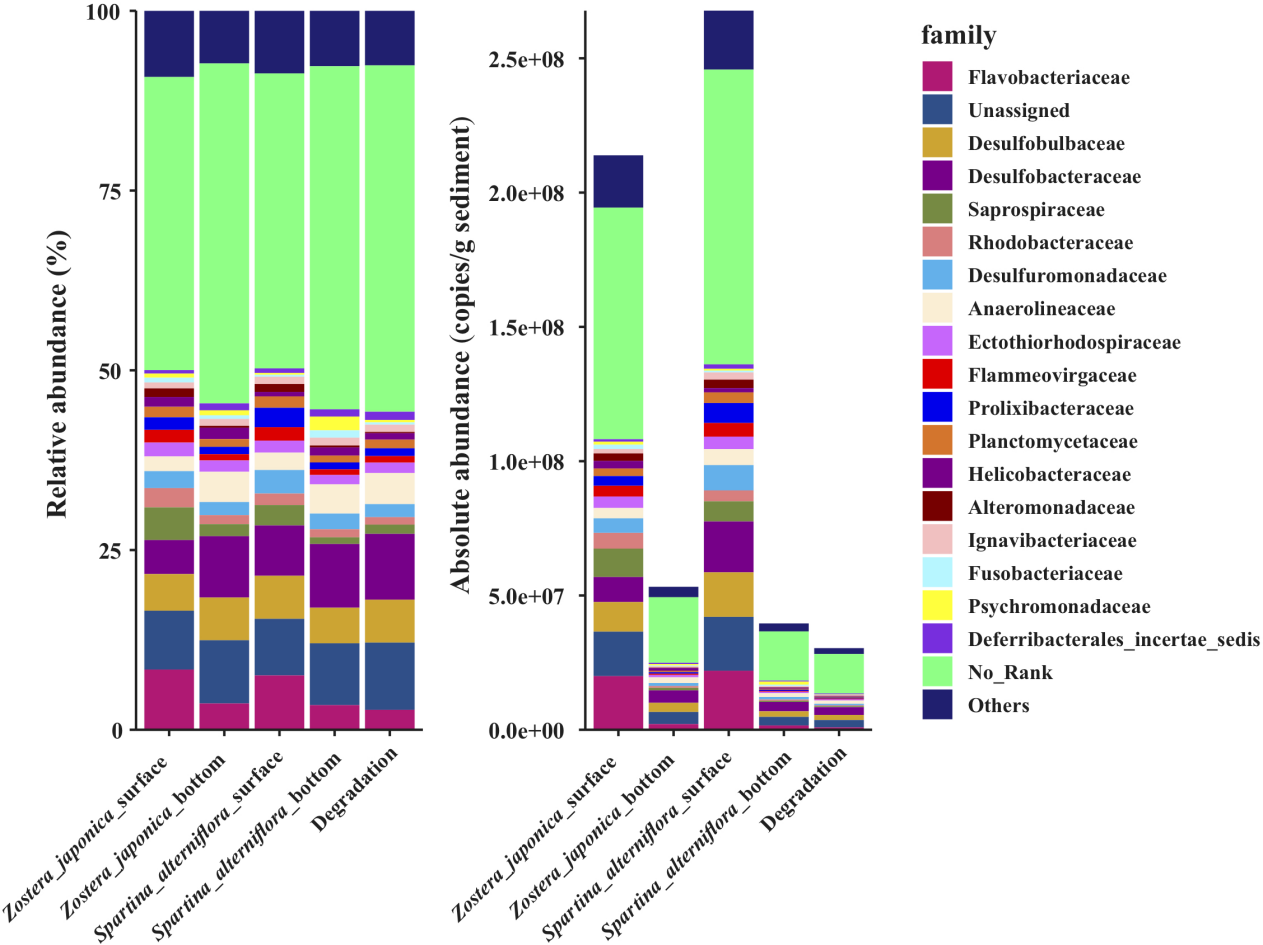
The relative and absolute abundances of the major bacteria at the family level. Absolute abundances (16S rRNA gene copies per g of sediment) and relative abundances (%) of the major bacterial family present in all the sediment samples

The relative abundances at the phylum level and the absolute copy number in the equal amount sample of different groups are shown in Figure 2. The results revealed that the absolute abundances of the surface samples of both *Z*. *japonica* and *S*. *alterniflora*, were significantly (*p *< .05) higher than those of the bottom samples (Appendix S1: Table A1). The absolute abundance of bacteria showed a 6.76‐fold in the *S*. *alterniflora* surface group compared to the *S*. *alterniflora* bottom group, and the absolute abundance of bacteria showed a 4.02‐fold in the *Z*. *japonica* surface group compared to the *Z*. *japonica* bottom group (Figure 2 and Appendix S1: Table A1). The absolute bacterial abundance of the degradation sample was lower than that of all the other samples, indicating that the coverage of plants could increase the bacterial abundance. In addition, the total abundance of bacteria showed a 1.25‐fold growth in the *S*. *alterniflora* surface group compared with the *Z*. *japonica* surface group (Figure 2 and Appendix S1: Table A1). Proteobacteria, Bacteroidetes, Planctomycetes, Acidobacteria, Chloroflexi, Actinobacteria, and Verrucomicrobia were the dominant phyla in all the samples (Figure 2). At the family level, Flavobacteriaceae, Desulfobulbaceae, Desulfobacteraceae, Saprospiraceae, Rhodobacteraceae, Desulfuromonadaceae, Anaerolineaceae, and Planctomycetaceae were the dominant families in all the samples (Figure 3).

### 3.3 Bacterial groups with significant differences

**FIGURE 4:**
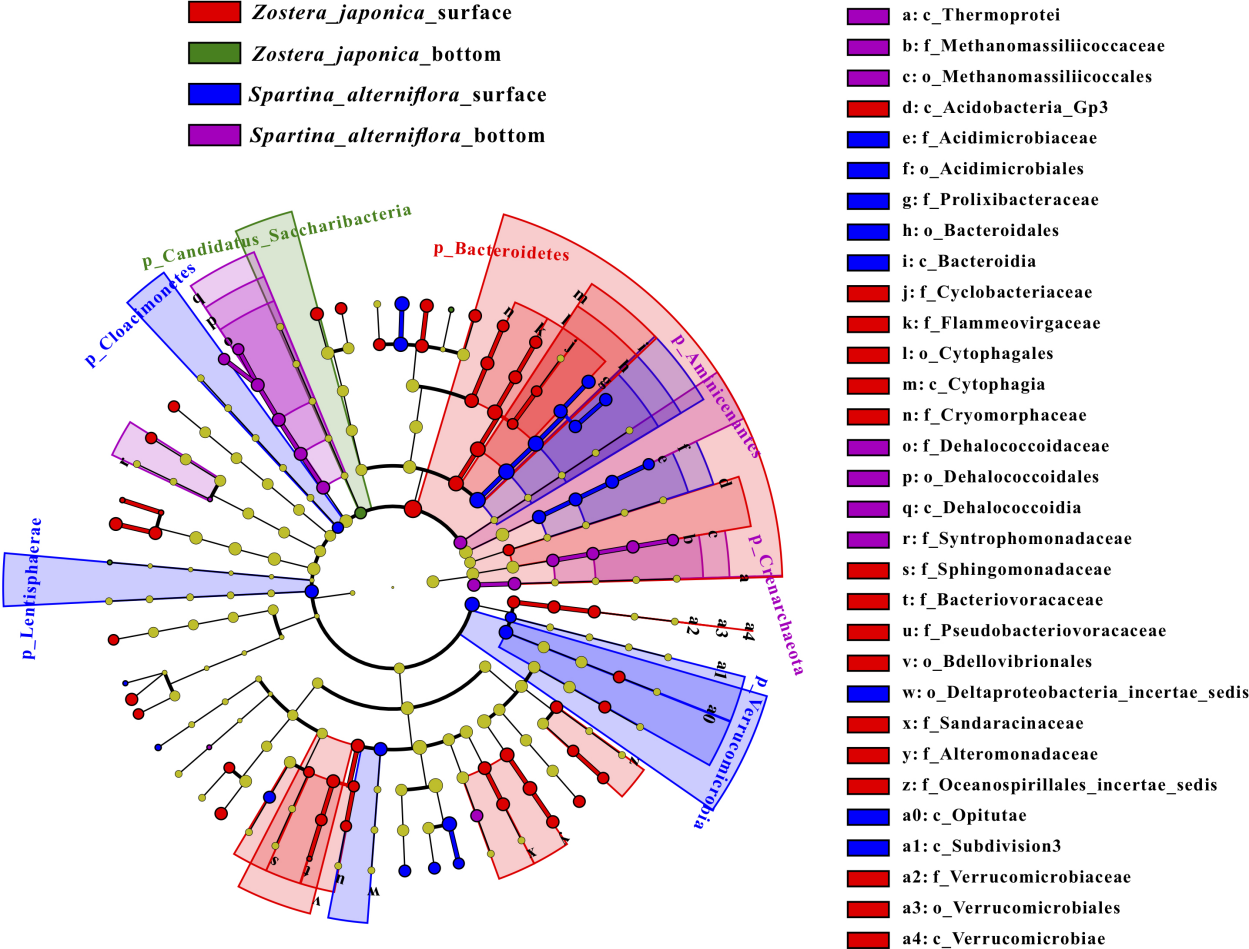
The output of the LEfSe algorithm, which identifies taxonomically consistent differences between *Z*. *japonica* surface, *Z*. *japonica* bottom, *S*. *alterniflora* surface and *S*. *alterniflora* bottom community members, was visualized. Different colors represent different groups. For example, the red circle in the branch indicates the species with significantly high abundance in the red group. Taxa with nonsignificant differences are represented as yellow circles, and the diameters of the circles are proportional to absolute abundance

Thirty‐one phylogenetic units were identified as statistically significant (*p *< .05) discriminative for the four groups (Figure 4). Acidobacteria (e.g., Acidobacteria Gp3), Cytophagia of the family Cytophagales (e.g., Cyclobacteriaceae, Flammeovirgaceae), Flavobacteriia of the family Flavobacteriales (e.g., Cryomorphaceae), Alphaproteobacteria (e.g., Sphingomonadaceae), Oligoflexia (e.g., Bacteriovoracaceae, Pseudobacteriovoracaceae, Bdellovibrionales), Deltaproteobacteria (e.g., Sandaracinaceae), Gammaproteobacteria (e.g., Alteromonadaceae, Oceanospirillales) and Verrucomicrobiaceaewere characteristic community members of the *Z*. *japonica* surface group (Figure 4). Acidimicrobiia (e.g., Acidimicrobiaceae), Bacteroidia (e.g., Prolixibacteraceae, Bacteroidales), Deltaproteobacteria (e.g., Deltaproteobacteriaincertaesedis) and Verrucomicrobia (e.g., Opitutae, Verrucomicrobia subdivision 3) were distinctive features of the *S*. *alterniflora* surface group (Figure 4). Archaea (e.g., Thermoprotei and Methanomassiliicoccaceae) and some anaerobic bacteria (e.g., Dehalococcoidaceae and Syntrophomonadaceae) were characteristic community members of the *S*. *alterniflora*bottom group (Figure 4).

### 3.4 Variation in bacterial community function in response to *S. alterniflora* invasion

**FIGURE 5:**
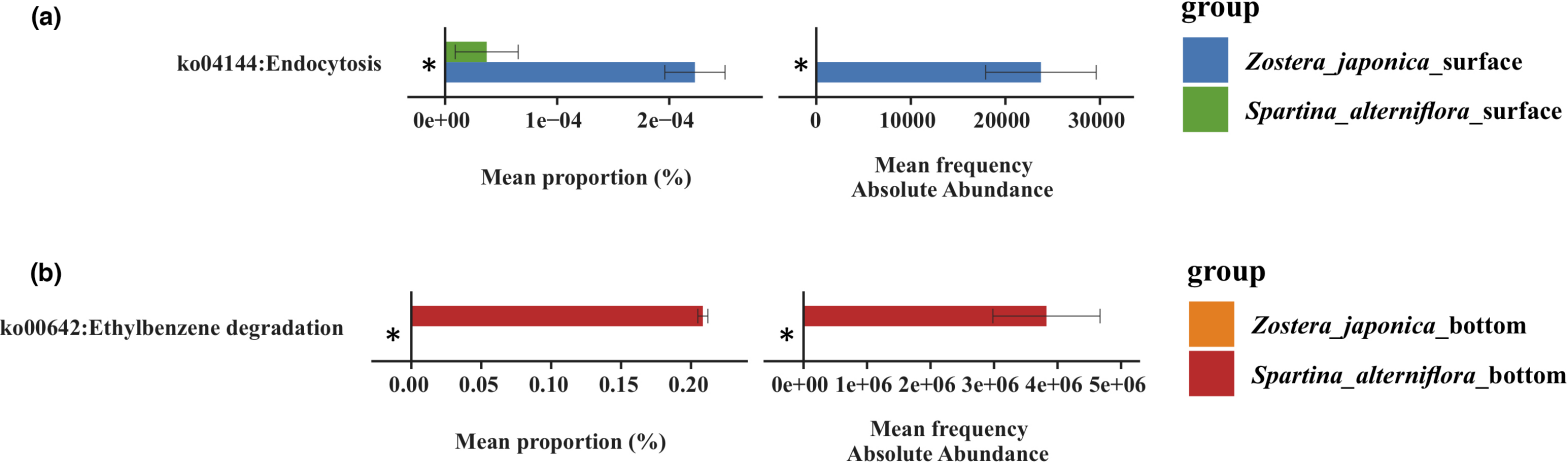
Different functions of the two quantitative methods compared with the bar chart. Different colors represent different groups of samples. The vertical coordinate is the functional pathway information, and the horizontal coordinate is the average relative abundance value (left: based on relative quantification (RQ)) and the average absolute abundance value (right: based on absolute quantification (AQ)). Data are expressed as mean ± SE (*p < .05 (Welch's *T*‐test) among the groups is shown in this figure). (a) The surface groups between *Z*. *japonica* and *S*. *alterniflora*; (b) the bottom groups between *Z*. *japonica* and *S*. *alterniflora*

The bacterial community function in the sediment was predicted by PICRUSt2, which revealed the variation in community function among the groups. For comprehensive analysis of the impact of *S*. *alterniflora* invasion on the bacterial community, Welch's *T* test was used to compare the functional abundance between *Z*. *japonica* and *S*. *alterniflora* samples. This analysis compared the *T* test results of functional prediction of relative quantification (RQ) and absolute quantification (AQ) and found significant differences among the groups. One function that differed significantly between the comparison of the surface and bottom groups of the two plants was screened out. They are respectively endocytosis pathway and Ethylbenzene degradation pathway (Figure 5).

Interestingly, the results showed that the *Z*. *japonica* surface group was significantly (*p *< .05) higher than the *S*. *alterniflora* surface group in endocytosis pathway, while the *S*. *alterniflora* bottom group was significantly (*p *< .05) higher than the *Z*. *japonica* bottom group in Ethylbenzene degradation pathway (Figure 5).

### 3.5 Correlation between sediment bacterial and physicochemical properties

The NMDS analysis showed that the bacterial communities in the *Z*. *japonica* surface and *S*. *alterniflora* surface samples were separated from those in the *Z*. *japonica* bottom, *S*. *alterniflora* bottom and degradation samples (Appendix S1: Figure A3), illustrating the difference in bacterial communities among the different group samples. The bacterial community in the degradation sample was separated from the *S*. *alterniflora* bottom group, but the *Z*. *japonica* group were mixed with the degradation group (Appendix S1: Figure A3). It showed that the plant effect of *S*. *alterniflora* is greater than that of *Z*. *japonica*.

**FIGURE 6:**
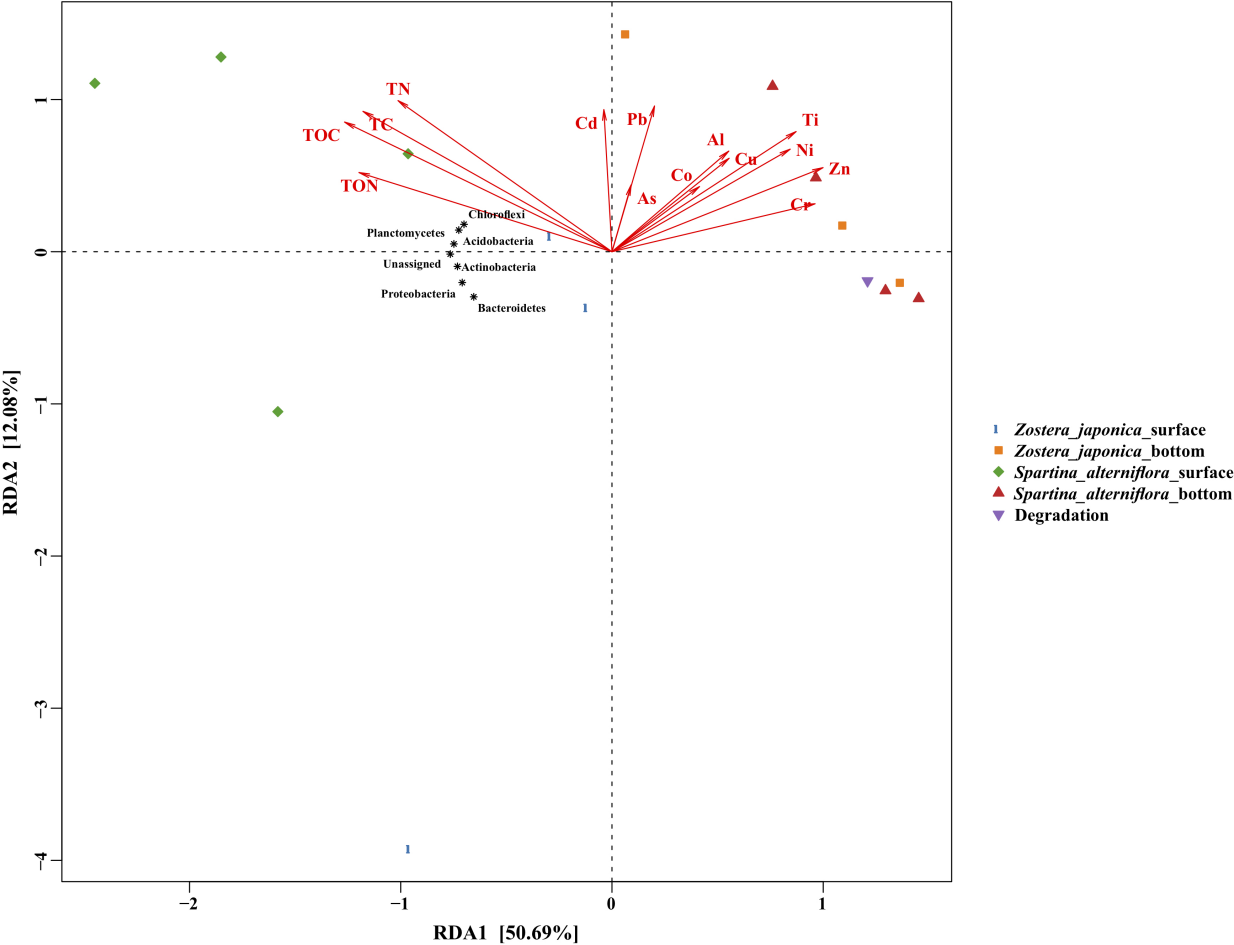
Redundancy analysis (RDA) diagram illustrating the relationships between the compositions of sediment bacterial communities at the phylum level from different groups under variable environments. Pb (Cr, Co, Ni, Cu, Zn, As, Cd, Al, Ti, V, Mn, Fe), the heavy mental concentrations in sediment; TC, total carbon; TN, total nitrogen; TOC, total organic carbon; TON, total organic nitrogen

To identify the relationship between the bacterial community composition and the physicochemical properties of the sediment samples, RDA (Figure 6) and Spearman correlation analysis (Appendix S1: Figure A4) were conducted. The first component (RDA1) separated the bottom (*Z*. *japonica* bottom and *S*. *alterniflora* bottom) and degradation samples from the surface samples (*Z*. *japonica* surface and *S*. *alterniflora* surface) and explained 50.69% of the variation. The variation was explained by the second component (RDA2) (Figure 6). Most of the heavy metals were negatively correlated with most of the dominant phyla. In contrast, the other five sediment physicochemical properties (including TC, TOC, TN, TON and the particle size distribution D50) were found to be positively (*p *< .05) correlated with the bacterial abundance (Figure 6 and Appendix S1: Figure A4). Interestingly, the heavy metal had a stronger effect on driving the bacterial communities in such lower abundance phyla, such as Fusobacteria, Marinimicrobia, and Tenericutes (Appendix S1: Figure A4). However, the other five sediment physicochemical properties had an intensive effect on driving the bacterial communities in the higher abundance phyla, such as Gemmatimonadetes and Acidobacteria (Appendix S1: Figure A4).

## 4 DISCUSSION

### 4.1 Effects of *S. alterniflora* invasion on sediment physicochemical properties

Plant species exert an essential role in regulating sediment physicochemical properties (Moreau et al., 2015). With the presence of *S*. *alterniflora*, soil physicochemical properties changed with increasing invasion age (Zhang, He, et al., 2019). However, the results of this research showed that the physicochemical properties of samples in different groups were not significantly different, especially the values of the TOC%, TC%, TON%, and TN% and the Cd and Mn concentrations (Table 1). This may be explained by the *S*. *alterniflora* samples collected, in which the invasion time was relatively short.

Sediment quality guidelines (SQGs) are commonly used to assess biologically adverse risks in marine sediments. Based on SQGs, the concentrations of trace elements in sediment were divided into three ranges (rare, occasional, and frequent adverse effects) as defined by the values of the threshold effect level (TEL) and probable effect level (PEL) (MacDonald et al., 2000). Compared with the TEL‐PEL SQGs (Appendix S1: Table A2), none of the metals in this study exceeded the PEL, suggesting that the levels of heavy metals in the sediment did not reach toxic standards. The values of As and Ni were in the range of TEL and PEL of the sample, indicating that the As and Ni had an occasional adverse effect. Furthermore, in comparison with the results from Weihai and Dalian seagrass bed habitats (Appendix S1: Table A2), all‐metal concentrations in this study ranked at high levels (Liu et al., 2019). Compared with the coasts of Thrace (apart from some samples of Pb and Cr), all‐metal concentrations in this study were at higher levels (Malea et al., 2019). These results revealed that the concentrations of heavy metals in this study were relatively high for *Z*. *japonica* and *S*. *alterniflora*, especially the As and Ni concentrations. *Z*. *japonica* could undertake and store heavy metals from sediment in the Yellow River Estuary. Lin's research (2016) showed that the concentrations of heavy metals were 1.00–2.03 times higher in seagrass‐rooted sediment than in adjacent nonseagrass sediment. However, in this study, most of the heavy metals in the degradation areas were higher than those in the regions with plant cover (whether *Z*. *japonica* or *S*. *alterniflora* was present). We speculated that after the degeneration of *Z*. *japonica*, the heavy metals absorbed by the plant also accumulated in the sediment, hence, their content was higher than that in the plant‐covered area. In addition, the concentrations of most heavy metals in *S*. *alterniflora* were higher than those in *Z*. *japonica* sediment samples (Table 1). Many species exerted equally as good bioindicators of marine pollution, showing significant correlations with the levels of trace elements in the surrounding environment (Bonanno et al., 2020; Malea et al., 2019). We assumed that the heavy metal concentrations in different plant sediment samples could reflect the heavy mental concentrations of plants themselves. Therefore, *S*. *alterniflora* may take up more stress from the heavy metals than *Z*. *japonica*.

### 4.2 The increased total abundances of the bacterial communities by *S. alterniflora* invasion via absolute quantification

Most of the previous studies on *S*. *alterniflora* invasion did not stratify the sediment (Nie et al., 2009; Yang et al., 2016, 2020; Zheng et al., 2019). In this study, we performed a comparative analysis between the surface sediment and the bottom sediment. The results showed that the bottom sediment was more similar to the degradation area (Figure 2), and their absolute quantities of bacteria were also of the same order of magnitude. In addition, the absolute number of bacteria in the surface sediment was significantly (*p *< .001) higher than that in the bottom and degradation areas (Figure 2 and Appendix S1: Table A1). It should be noted that all the significance analyses of the degradation group were not included because there was only one sample.

Many studies have shown that the invasion of *S*. *alterniflora* can change the composition and structure of the microbial communities in the invasion site (Yang et al., 2020; Zhang et al., 2011; Zheng et al., 2019). However, few studies have committed to addressing the absolute abundances of bacterial taxa. The interpretation of bacterial community dynamics calculated solely from relative abundance may be misleading. Once the total abundance is not fixed, fluctuations in the absolute abundance of a particular taxon may not generate a significant change in the calculated relative abundance. In this study, an absolute quantification 16S rRNA sequencing method was adopted to investigate the bacterial community abundances. As confirmed by previous studies (Smets et al., 2016), the relative abundance can reflect the sensitivity and the growth rate of the dominant species in a single bacterial community, while the absolute abundance can uncover the comprehensive dynamics of different bacterial communities. Thus, using the absolute quantification 16S rRNA sequencing method allows us to compare differentially abundant taxa across different group samples and widely offers more detailed insights into bacterial community dynamics.

The stem density, height, and total biomass of *Z*. *japonica* decreased to different degrees after the invasion of *S*. *alterniflora* (Ma et al., 2020). In this study, it was found that the absolute quantity of bacteria in the surface samples of *S*. *alterniflora* was higher than that of *Z*. *japonica*. This may be the reason for the better adaptation and eventual success of the invasion.

In this study, RDA analyses clearly indicated that variations in the composition of sediment bacterial communities at the phylum level were the most intimately related to TOC (Figure 6), which further verified that TOC was a driving factor for the changes in the sediment bacterial communities (Yang et al., 2020). In addition, the composition of soil bacterial communities was highly associated with the concentrations of most heavy metals (Figure 6). Soil salinity (Rath et al., 2019) and soil pH (Bainard et al., 2016; Rousk et al., 2010; Yang et al., 2020) appeared to be the primary drivers for the composition of soil bacterial communities. In this study, there was no difference in the sediment pH. All the sediment samples which sampled from the same intertidal zone were subjected to frequent inundation by semidiurnal tides.

At the family level, Flavobacteriaceae was one of the dominant families in all the samples, especially in the surface sediment (Figure 3). Flavobacteriaceae is a family known for polysaccharide and peptide degradation. Because of its adaptation to diverse ecological niches, Flavobacteriaceaeexhibits high genomic plasticity and potential ecological transitions (Zhang, Yoshizawa, et al., 2019). Further study is needed on the evolution of Flavobacteriaceae in the process of *S*. *alterniflora* invasion. Additionally, sulfate‐reducing bacteria were enriched in all the samples, including Desulfobulbaceae, Desulfuromonadaceaeand Desulfobacteraceae (Figure 3), which indicated that sediment microorganisms play an essential role in the sulfur cycle. Interestingly, the bacteria in the surface sediments were more abundant than those in the bottom sediments (Figure 2 and Appendix S1: Table A1). Trace metals that originate from natural and anthropogenic sources are deposited into the bottom sediments (Zhang, Bai, et al., 2019; Zhang, He, et al., 2019; Zhang, Yoshizawa, et al., 2019). In addition, RDA analysis indicated that most of the heavy metals were negatively correlated with most of the dominant phyla. Li's research (2017) showed that the change in microbial community composition was a result of many factors including metal contents and othermetal contamination accompanied pH, carbon and nitrogen change. Among the factors, heavy metals were the most important factor affecting microbes. Therefore, we speculate that the content of heavy metals can reduce the abundance of bacteria.

### 4.3 Indicator bacterial communities in *S. alterniflora* and their ecological effects

LEfSe analysis revealed the significant enrichment of Bacteroidia and Acidimicrobiaceae in the *S*. *alterniflora* surface sediment relative to the other groups of samples (Figure 4). The increased abundance of Bacteroidetes following *S*. *alterniflora* invasion can enhance the degradation of refractory *S*. *alterniflora* residues and promote soil organic carbon and soil organic nitrogen sequestration (Yin et al., 2020). In this study, we found the *Z*. *japonica* surface group also enriched Bacteroidetes (Cytophagia and Flavobacteriia were included, and Bacteroidia was not included) (Figure 4).

Acidimicrobiaceae might play a key role in this anaerobic biological process that uses ferric iron as an electron acceptor while oxidizing ammonium to nitrite. After ammonium was oxidized to nitrite, nitrogen loss proceeded via denitrification and/or anammox (Huang & Jaffé, 2015). Iron ammoxidation plays an important role in the circulation of nitrogen in the environmental system and has a potential functional role in alleviating nitrogen pollution in the environment. In this study, LEfSe analysis was not enriched in the *Z*. *japonica* bottom group while the *S*. *alterniflora* bottom group enriched some bacteria that may be dominant for *S*. *alterniflora* invasion (Figure 4). For example, Dehalococcoidaceaemay be the main HCB‐dechlorinating bacteria in the soil (Song et al., 2017). Acidobacteria are considered as slow growing oligotrophic groups that thrive in soils with a low availability of resources (Pascault et al., 2013). The greatly decreased relative abundances of Acidobacteria in the *Z*. *japonica* surfacesediment likely resulted from the lowest sediment nutrient substrate levels. It is presumed that the higher abundance of dominant groups (such as Bacteroidia, Acidimicrobiaceae, and Dehalococcoidaceae) in *S*. *alterniflora* sediment can enhance the root growth of *S*. *alterniflor* to better adapt to the environment to achieve successful invasion. This supports our hypothesis that some dominant groups could better adapt to the environment and able to accelerate the invasion of local plants. In addition, Welch's *T*‐test showed that the *S*. *alterniflora* bottom group was significantly higher than the *Z*. *japonica* bottom group in Ethylbenzene degradation pathway (Figure 5). Ethylbenzene is toxic to plants and affects the removal efficiency (Sriprapat et al., 2014). The pathogenic invasion reduces microbial diversity and abundance in the rhizosphere (Wei et al., 2018). Microorganisms on leaves and soil may help to degrade Ethylbenzene for plant's.

growth metabolism (Mukhtar et al., 2011). This indicated that compared with *Z*. *japonica*, the bacterial community of *S*. *alterniflora* may help to degrade some toxic exogenous substances (e.g., the heavy metals) and can better adapted to the environment, which is conducive to colonization.

## 5 CONCLUSIONS

This study investigated the alterations in sediment bacterial communities and inferred the deterministic processes driving these variations along with *S*. *alterniflora* invasion on *Z*. *japonica* in the Yellow River Estuary. We found that the absolute quantity of bacteria in the surface layer was significantly higher than that in the bottom and degradation areas. The content of heavy metals could reduce the abundance of bacteria. *S*. *alterniflora* invasion increased the bacterial community abundances in surface sediment samples in comparison with degradation and native plant communities. Flavobacteriaceae had the highest bacterial abundance in most of the samples. In addition, the dominant groups (such as Bacteroidia, Acidimicrobiaceae, and Dehalococcoidaceae) in *S*. *alterniflora* sediment can better adapt to the environment. This study provides information to understand the effects of *S*. *alterniflora* invasion of the *Z*. *japonica* sediment bacterial community, which is helpful to clarify the potential invasion mechanism of *S*. *alterniflora*on *Z*. *japonica*from the perspective of microbiome level.

## AUTHOR CONTRIBUTIONS

**Zenglei Song:** Conceptualization (lead); Formal analysis (equal); Investigation (equal); Validation (equal); Writing – original draft (lead). **Yanyu Sun:** Conceptualization (supporting); Formal analysis (supporting); Investigation (equal). **Pengyuan Liu:** Resources (lead); Software (equal). **Yibo Wang:** Software (equal); Supervision (supporting); Writing – review & editing (equal). **Yanyan Huang:** Investigation (equal); Software (supporting). **Yan Gao:** Supervision (supporting); Writing – review & editing (equal). **Xiaoke Hu:** Funding acquisition (lead); Supervision (lead); Writing – review & editing (equal).

## CONFLICT OF INTEREST

The authors declare no conflict of interest.

## Supporting information

Appendix S1

## Data Availability

The data that support the findings of this study are openly available in NCBI Sequence Read Archive (SRA) database under accession number “PRJNA641753” (https://www.ncbi.nlm.nih.gov/bioproject/PRJNA641753/). GenBank accessions: SRX8613158–SRX8613172.
