# Supplementary material for: Invasion of Spartina alterniflora on Zostera japonica enhances the abundances of bacteria by absolute quantification sequencing analysis
Source: Ecol Evol. 2022 May 19;12(5):e8939. doi: 10.1002/ece3.8939 (PMC9120208; doi:10.1002/ece3.8939)
Supplement: Supplementary file 1 — Appendix S1 [file ECE3-12-e8939-s001.doc]

**Appendix**

**Table A1.** The absolute abundances of the sediment samples.

| Group Name | Absolute abundance (copies/g sediment) |
| --- | --- |
| *Z. japonica* surface (n=3) | (2.14 ± 0.43) E + 08 **^a^** |
| *Z. japonica* bottom (n=3) | (5.32 ± 2.04) E + 07 **^b^** |
| *S. alterniflora* surface (n=4) | (2.68 ± 0.24) E + 08 **^a^** |
| *S. alterniflora* bottom (n=4) | (3.96 ± 1.08) E + 07 **^b^** |
| Degradation (n=1) | 3.04 E + 07 |
| *P* value | 0.029 |

Data are expressed as mean ± SE (degradation group was not included). *P* value (Kruskal-Wallis test). Different superscript lowercase letters indicate statistically significant differences at the α = 0.05 level among the sediment groups, using Tukey’s honestly significant difference test (degradation group was not included).

**Table A2.** Concentrations (mg/kg) of heavy metals in the sediment samples (*Z. japonica* habitat) compared to those in the study area.

| Heavy mental | Study site/Reference | | | | TEL^a^ | PEL^a^ |
| --- | --- | --- | --- | --- | --- | --- |
|  | Yellow River Estuary | Weihai | Dalian | Coasts of Thrace |  |  |
|  | This study | (Liu et al., 2019) | (Liu et al., 2019) | (Malea et al., 2019) |  |  |
| As | 8.75-11.61 | ~3.17 | ~2.81 | No data | 7.24 | 41.6 |
| Pb | 11.34-16.41 | ~5.60 | ~4.26 | 24.55-47.67 | 30.2 | 112 |
| Cd | 0.26-0.31 | ~0.10 | ~0.05 | 0.081-0.752 | 0.68 | 4.21 |
| Cr | 4.4-9.21 | ~2.76 | ~2.00 | 1.65-14.93 | 52.3 | 160 |
| Cu | 9.51-16.03 | ~4.09 | ~2.29 | 2.74-9.64 | 18.7 | 108 |
| Zn | 22.5-41.41 | ~11.39 | ~9.26 | No data | 124 | 271 |
| Ni | 12.32-19.94 | ~3.80 | ~2.07 | 4.66-16.31 | 15.9 | 42.8 |
| Co | 4.06-6.56 | ~1.42 | ~1.21 | 2.08-4.17 | No data | No data |

^a^ TEL: threshold effect level; PEL: probable effect level (MacDonald et al., 2000)


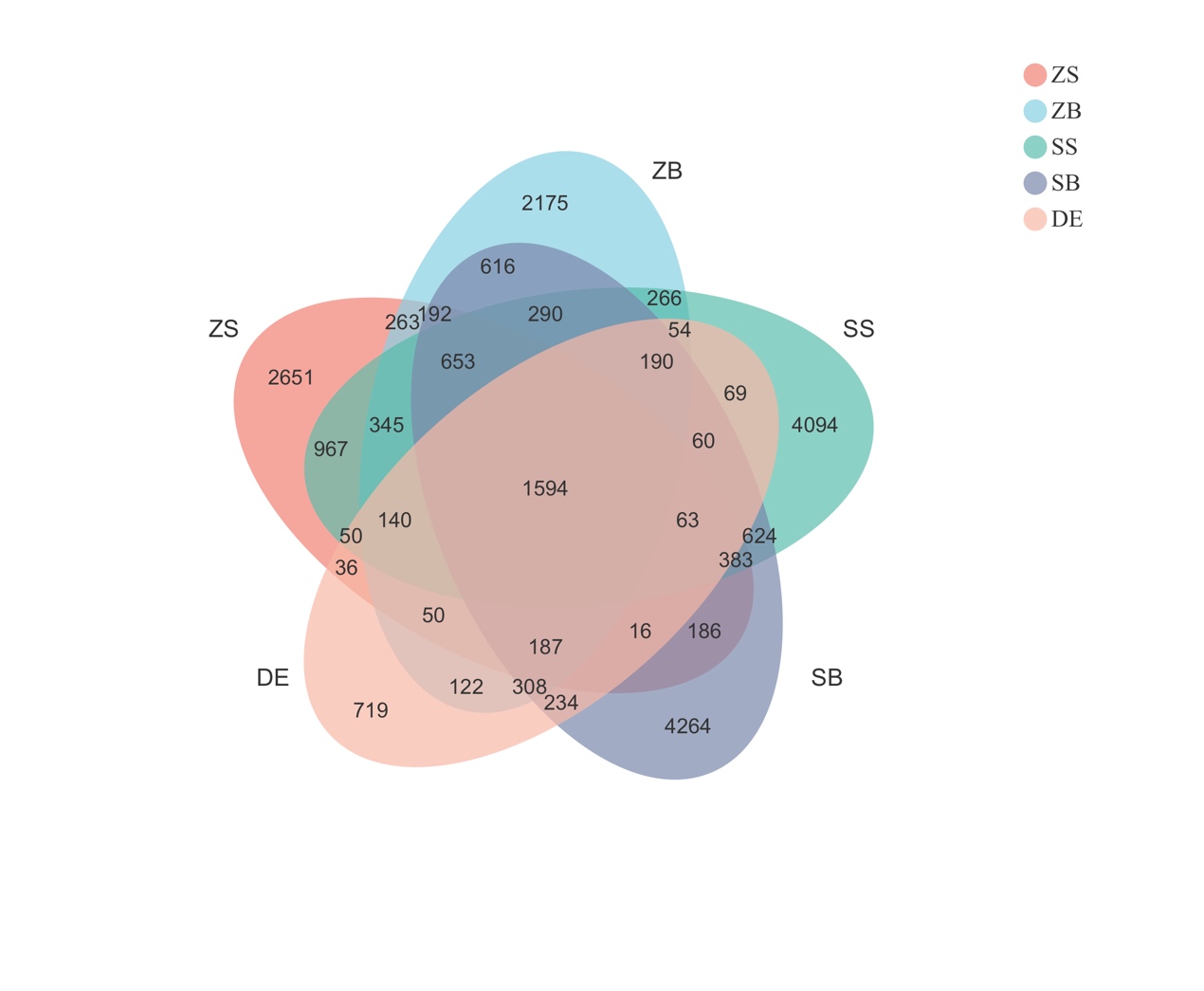


**Fig. A1.** Venn map of different groups based on the ASVs. ZS: *Z. japonica* surface; ZB: *Z. japonica* bottom; SS: *S. alterniflora* surface; SB: *S. alterniflora* bottom; DE: degradation.


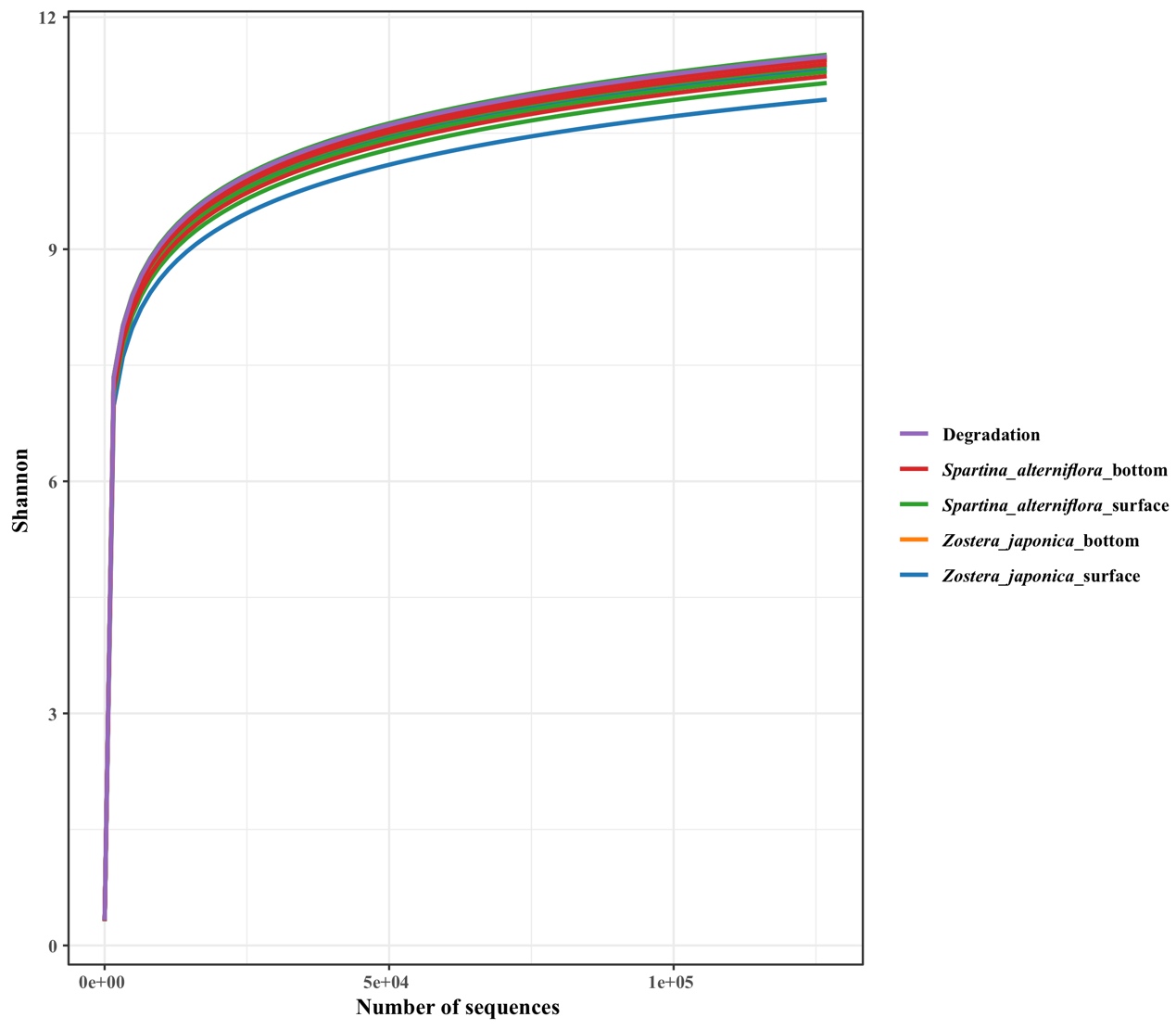


**Fig. A2.** Rarefaction curves of the sediment samples, relating the sequencing effort with an estimate of the number of microbial species, as inferred by the number of ASVs.


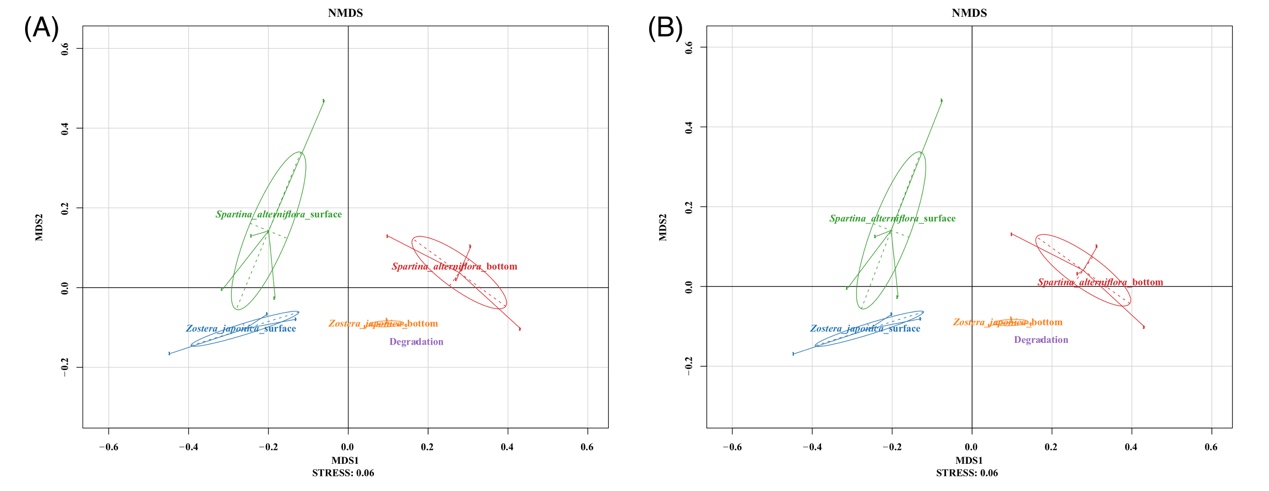


**Fig. A3.** The Nonmetric multidimensional scaling (NMDS) analysis using Jaccard distance. A based on absolute quantification (AQ) and B based on relative quantification (RQ).


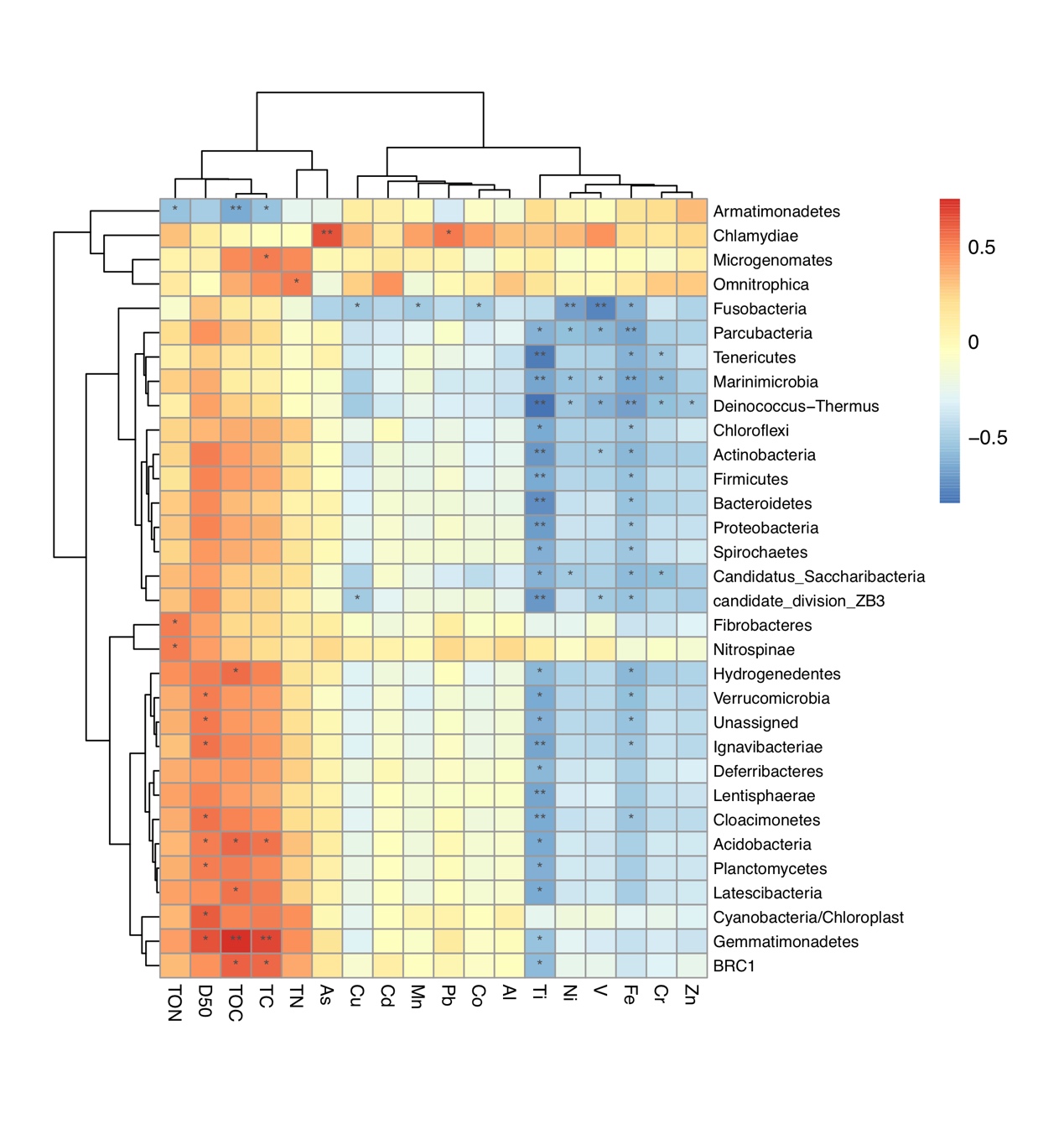


**Fig. A4.** Spearman correlation analyses show the absolute abundances of bacterial phyla that are significantly positively/negatively correlated with heavy metals and physicochemical parameters in the sediment. The right side of the legend is the color range of varying r values, with *p* < 0.05* and *p* < 0.01**. TON: total organic nitrogen; TOC: total organic carbon; TN: total nitrogen; TC: total carbon; Pb (Cr, Co, Ni, Cu, Zn, As, Cd, Al, Ti, V, Mn, Fe): the heavy mental concentrations in sediment; D50: the median diameter or the medium value of the particle size distribution.
